# A Computational Pipeline for High- Throughput Discovery of *cis*-Regulatory Noncoding RNA in Prokaryotes

**DOI:** 10.1371/journal.pcbi.0030126

**Published:** 2007-07-06

**Authors:** Zizhen Yao, Jeffrey Barrick, Zasha Weinberg, Shane Neph, Ronald Breaker, Martin Tompa, Walter L Ruzzo

**Affiliations:** 1 Department of Computer Science and Engineering, University of Washington, Seattle, Washington, United States of America; 2 Department of Molecular Biophysics and Biochemistry, Yale University, New Haven, Connecticut, United States of America; 3 Department of Molecular, Cellular, and Developmental Biology, Yale University, New Haven, Connecticut, United States of America; 4 Department of Genome Sciences, University of Washington, Seattle, Washington, United States of America; 5 Howard Hughes Medical Institute, Yale University, New Haven, Connecticut, United States of America; Washington University in St. Louis, United States of America

## Abstract

Noncoding RNAs (ncRNAs) are important functional RNAs that do not code for proteins. We present a highly efficient computational pipeline for discovering *cis*-regulatory ncRNA motifs de novo. The pipeline differs from previous methods in that it is structure-oriented, does not require a multiple-sequence alignment as input, and is capable of detecting RNA motifs with low sequence conservation. We also integrate RNA motif prediction with RNA homolog search, which improves the quality of the RNA motifs significantly. Here, we report the results of applying this pipeline to Firmicute bacteria. Our top-ranking motifs include most known Firmicute elements found in the RNA family database (Rfam). Comparing our motif models with Rfam's hand-curated motif models, we achieve high accuracy in both membership prediction and base-pair–level secondary structure prediction (at least 75% average sensitivity and specificity on both tasks). Of the ncRNA candidates not in Rfam, we find compelling evidence that some of them are functional, and analyze several potential ribosomal protein leaders in depth.

## Author Summary

For decades, scientists believed that, with a few key exceptions, RNA played a secondary role in the cell. Recent discoveries have sharply revised this simple picture, revealing widespread, diverse, and surprisingly sophisticated roles for RNA. For example, many bacteria use RNA elements called “riboswitches” to switch various gene activities on or off in response to extremely sensitive detection of specific molecules. Discovery of new functional RNA elements remains a very challenging task, both computationally and experimentally. It is computationally difficult largely because of the importance of an RNA molecule's 3-D structure, and the fact that molecules with very different nucleotide sequences can fold into the same shape. In this paper, we propose a computational procedure, based on comparing the genomes of multiple bacteria, for discovery of novel RNAs. Unlike most previous approaches, ours does not require a letter-by-letter alignment of these diverse genomes, making it more applicable to RNA elements whose structure, but not nucleotide sequence, has been preserved through evolution. In an extensive test on the Firmicutes, a bacterial phylum containing well-studied organisms such as Bacillus subtilis and important pathogens such as anthrax, we recover most known noncoding RNA elements, as well as making many novel predictions.

## Introduction

Recent discoveries of novel noncoding RNAs (ncRNAs) such as microRNAs and riboswitches suggest that ncRNAs have important and diverse functional and regulatory roles that impact gene transcription, translation, localization, replication, and degradation [1–3]. In the last few years, several groups have performed genome-scale computational ncRNA predictions based on comparative genomic analysis. In particular, Barrick et al. [4] used a pairwise, BLAST-based approach to discover novel riboswitch candidates in bacterial genomes, many of which now have been experimentally verified. Similar studies have been conducted in various bacterial groups [5–8]. More recent work has extended these searches to eukaryotes [9–13], discovering a large number of known microRNAs while producing thousands of novel ncRNA candidates. With some exceptions, such as [4] and [13], these approaches follow a similar paradigm, which is to search for conserved secondary structures on multiple-sequence alignments that are constructed based on sequence similarity alone. Typically, these schemes use measures such as mutual information between pairs of alignment columns to signal base-paired regions. However, the signals such methods seek, namely compensatory base-pair mutations, are exactly the signals that may cause sequence-based alignment methods to misalign, or alternatively refuse to align, homologous ncRNA sequences. Even local misalignments may weaken this key structural signal, making the methods sensitive to alignment quality, which is especially problematic on diverged sequences.

In this paper, we present a novel structure-oriented computational pipeline for genome-scale prediction of *cis*-regulatory ncRNAs. It exploits, but does not require, sequence conservation. The pipeline differs from previous methods in three respects. First, it searches in unaligned upstream sequences of homologous genes, instead of well-aligned regions constructed by sequence-based methods. Second, we predict RNA motifs in unaligned sequences using a tool called CMfinder [14], which is very sensitive to RNA motifs with low sequence conservation, and robust to inclusion of long flanking regions or unrelated sequences. Finally, we integrate RNA motif prediction with RNA homology search. For every predicted motif, we scan a genome database for more homologs, which are then used to refine the model. This iterative process improves the model and expands the motif families automatically.

In this study, we apply this pipeline to discover ncRNA elements in prokaryotes. We chose prokaryotes mainly because of the large number of fully sequenced genomes and the great sequence divergence among the species, which can be well-exploited by our approach.

Our approach has two key advantages. First, it is efficient and highly automated. Earlier steps are more computationally efficient than later steps, and we can apply filters between steps so that poor candidates are eliminated from subsequent analysis. Thus, even though we use some computationally expensive algorithms, the pipeline is scalable to larger problems. Besides providing RNA motif prediction, the pipeline also integrates gene context and functional analysis, which facilitates manual biological evaluation. Second, this pipeline is highly accurate in finding prokaryotic ncRNAs, especially RNA *cis*-regulatory elements.

To demonstrate the performance of this approach, we report our search results in Firmicutes, a Gram-positive bacterial division that includes *Bacillus subtilis,* a relatively well-studied model organism with many known ncRNAs. The method exhibits low false-positive rates on negative controls (permuted alignments), and low false-negative rates on known Firmicute ncRNAs. The RNA family database (Rfam) [15], a partially hand-curated database of noncoding RNAs, includes 13 ncRNA families categorized as *cis-*regulatory elements with representatives in *B. subtilis.* Of these, 11 are included among our top 50 predictions and a 12th appears somewhat lower in our ranking. Two other Rfam families are also represented among our top 50 predictions. In addition, both the secondary structure prediction and identified family members are in excellent agreement with Rfam annotation. For the 14 Rfam families mentioned above, we achieved 91% specificity and 84% sensitivity on average in identifying family members, and 77% specificity and 75% sensitivity in secondary structure prediction. Many promising novel ncRNA candidates were also discovered and are discussed below.

## Results

**Figure 1:**
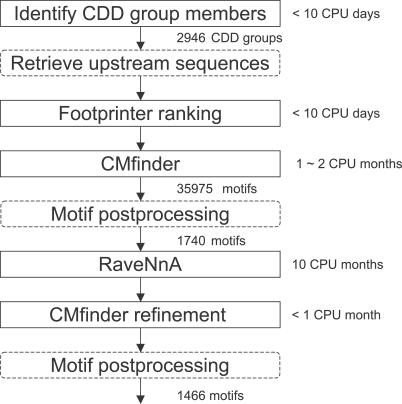
Pipeline Flowchart The boxes with solid lines indicate steps involving intensive computation (approximate running time is specified next to each). Other intermediate steps are specified in the boxes with dashed lines.

In outline, our pipeline consists of the following major steps. (See Figure 1, Materials and Methods, and the online supplement at http://bio.cs.washington.edu/supplements/yzizhen/pipeline for more details.) First, we used the National Center for Biotechnology Information's (NCBI's) Conserved Domain Database (CDD) [16] to identify homologous gene sets. For each gene, we collected its 5′ upstream sequence. We call the set of 5′ sequences associated with one CDD group a dataset. *cis*-Regulatory elements are often conserved within such groups. Second, we applied FootPrinter [17], a DNA phylogenetic footprinting tool, to select datasets that are likely to host ncRNAs. In our experience, functional RNAs such as riboswitches often show low overall sequence conservation, but contain interspersed patches where conservation is high. FootPrinter is very effective at highlighting the latter regions. Third, we used CMfinder to infer RNA motifs in each unaligned sequence dataset. CMfinder is a structure-oriented local alignment tool that is robust to varying sequence conservation and length of extraneous flanking regions. We postprocessed motifs to identify distinct motifs corresponding to different RNA elements by removing poor and redundant motifs and clustering the rest based on overlap. Fourth, we used RaveNnA [18–20] to find additional motif instances by scanning the prokaryotic genome database. Riboswitches, for example, often regulate multiple operons that contribute to a single pathway, but no single CDD domain will be common to all of these operons. Thus, the search step was a powerful adjunct to the motif discovery process. These newly discovered motif members were incorporated into a refined motif model, again using CMfinder, and in some cases the search and motif refinement steps were repeated. Motif postprocessing was also repeated after the search/refinement steps. Both CMfinder and RaveNnA rely on the Infernal covariance model software package [21] for RNA motif modeling and search. Finally, we performed gene context analysis and literature searches (manually) for the top-ranking motifs.

We included 44 completely sequenced Firmicute species (see the online supplement at http://bio.cs.washington.edu/supplements/yzizhen/pipeline) and 2,946 CDD groups in this study. For each of the three main steps—FootPrinter, CMfinder, and RaveNnA-based refinement—we produced scores to determine which candidates were worthy of continuing analysis. For evaluation purposes, we recorded the scores of candidates at each step, but eliminated none; in the future, we will use them as filters.

The initial CMfinder step produced 35,975 motifs in total. Motif postprocessing reduced this to 1,740 motifs grouped into 1,050 clusters. After RaveNnA-based refinement, more motifs were identified as redundant and removed. A total of 1,466 motifs remained, grouped into 1,060 clusters. (A few of the original clusters were subdivided based on divergent search results.) The full list of candidates is available in the online supplement at http://bio.cs.washington.edu/supplements/yzizhen/pipeline.

### Negative Controls: Permuted Alignments

**Figure 2:**
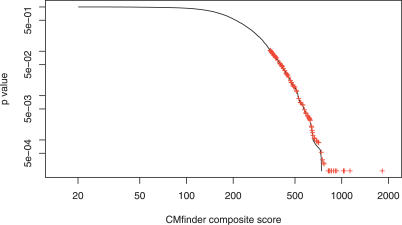
The Empirical *p*-Value Distribution Based on the Permutation Test The black curve shows the complementary cumulative distribution function for the composite scores on randomized datasets (i.e., for each score, the fraction of permuted alignments exceeding that score). The red pluses show the *p*-values for the composite scores of the motifs in the original (unpermuted) datasets. All *p*-values are greater than or equal to 2 × 10^−4^ as there are only 5,000 samples in the background distribution.

To evaluate how many of our top candidates could have arisen by chance, we performed a randomized control experiment. We first computed CLUSTALW alignments of the 100 sequence datasets having the highest motif scores (before the RaveNnA scan). We then randomly permuted the alignments 50 times, maintaining the approximate gap pattern (see the online supplement at http://bio.cs.washington.edu/supplements/yzizhen/pipeline). After degapping each permuted alignment (treating it as a set of unaligned sequences), we applied CMfinder, retaining the top-ranking motif from each randomized dataset. We used this collection of 5,000 motifs to estimate the background score distribution, and to infer *p*-values for predicted motifs in the original datasets. Results are shown in Figure 2. By this measure, all 100 top-scoring motifs have *p*-values less than 0.1, with the median at 0.016. In addition, 73 of the 100 candidates in the original dataset score higher than all motifs in the corresponding randomized datasets.

Note that this estimation of *p*-values is imperfect. In particular, with the scoring scheme we used, datasets containing phylogenetically close sequences tend to score well in comparison to more diverged sets, because permuting the CLUSTALW alignments preserves their sequence conservation. (Independently permuting individual sequences instead of alignments would be less realistic, since in practice *cis*-regulatory RNA motifs are often embedded in regions exhibiting some sequence conservation for other reasons.) Although imperfect, the significance of real motifs tends to be underestimated by this method.

### Positive Controls: Discovering Known Rfam Families

To roughly assess the sensitivity with which the method discovers true ncRNAs, we looked at its recovery of known Rfam (version 7.0) families. We masked matches to Rfam's tRNA and rRNA models, since otherwise these widespread, strong motifs might hide nearby, weaker, but still interesting ncRNA structures. Other Rfam families were not masked and serve as a positive control for our methods. Table 1 shows the distribution of known Rfam families in our candidate list, together with their ranks after running FootPrinter, CMfinder, and RaveNnA. We used the refined motifs as the final output.

**Table 1:**
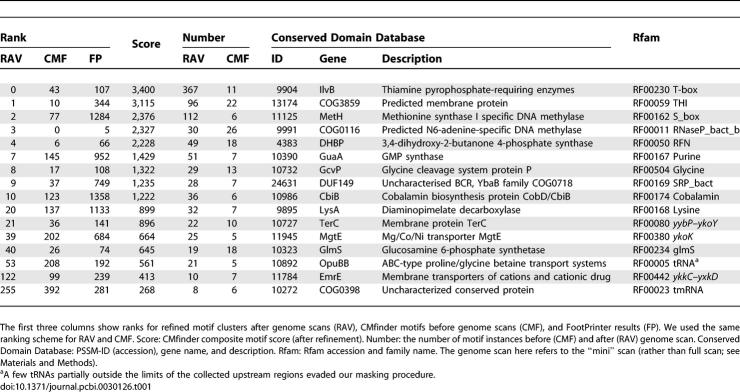
Motifs That Correspond to Rfam Families

According to Rfam, *B. subtilis* contains members of 21 families, categorized into 13 *cis*-regulatory families, one intron element, and seven RNA gene families. We masked tRNAs and rRNAs (four of the seven gene families). Of the 17 remaining families, 13 appear within our top 50 candidates: 11 *cis*-regulatory families present in *B. subtilis,* together with two of the gene families (RNaseP_bact_b and SRP_bact). The four families not represented are two *cis*-regulatory elements (*ykkC-yxkD* and *ydaO-yuaA*)*,* one RNA gene (tmRNA), and one intron element (Intron_gpI). The exclusion of Intron_gpI is not surprising, as we did not search intragenic regions. The *ydaO–yuaA* motif escaped detection because it is present in only three of the 68 sequences in its CDD group. The *ykkC–yxkD* and tmRNA motifs, although not among our top 50, would still have been ranked high enough to be discovered in a blind test. Note that, although our computational pipeline is oriented toward discovery of *cis*-regulatory elements, we sometimes find RNA genes such as RNaseP, SRP, and tmRNA because they happen to be conserved in synteny. We also found a partial tRNA motif, not masked since parts of the tRNA lie outside of the collected upstream sequences.

We can potentially filter the candidates at each step to scale this pipeline for larger genomes. In particular, we could have applied CMfinder to only the top half of the datasets according to FootPrinter, and performed genome scans on only the top 500 motifs, without missing any real Rfam families as listed in Table 1. On average, it takes FootPrinter less than 1 min, and CMfinder 10 min to process each dataset, while it takes RaveNnA 4.8 h to scan each motif. We could save considerable computation time by running expensive algorithms only on good candidates.

As shown in Table 1, the ranks for most known ncRNAs improve at each successive step of the pipeline, as more supporting evidence is found. Starting from FootPrinter motifs, CMfinder improves the alignment and identifies consensus secondary structure, while genome scans locate many more motif instances, typically providing still better alignments and additional clues to their functions.

To measure the quality of our automatically constructed motif models, we compared them with Rfam alignments for the same families. Rfam's covariance models are built from hand-curated “seed” alignments/structure annotations. These in turn are used to build Rfam's “full” alignments by automatically searching RFAMSEQ (http://www.sanger.ac.uk/Software/Rfam/ftp.shtml), a high-quality, nonredundant subset of EMBL (http://www.ebi.ac.uk/embl), and automatically aligning all hits.

For the 14 Rfam families in Table 1 for which we found good matching motifs, we selected the top two motifs from each family, and performed full-genome scans on RFAMSEQ, the same sequence database used to construct the Rfam full alignment. To reduce computation time, we did not scan eukaryote genomes, and the Rfam hits from these genomes were excluded from the following analysis. (This treatment affects only a few eukaryotic Cobalamin and Lysine hits, all believed to be Rfam errors or bacterial contamination in the genome sequences, plus a few THI hits, which are real.) For each motif, we selected scan hits at an E-value cutoff of 100, reconstructed the motif alignments using CMfinder, and removed the low-scoring instances (<20 bits). We compared these predicted motifs to corresponding Rfam full alignments, which serve as the gold standard in this test. Table 2 shows the accuracy of our motifs in membership prediction, motif coverage, and secondary structure prediction. Secondary structures were compared at the base-pair level, and only the base pairs with at least one end falling into the overlapped regions are counted. For both predicted motifs and Rfam full alignments, we removed noncanonical base pairs from each sequence. Of the two motifs chosen for each family, we report the one with better results.

**Table 2:**
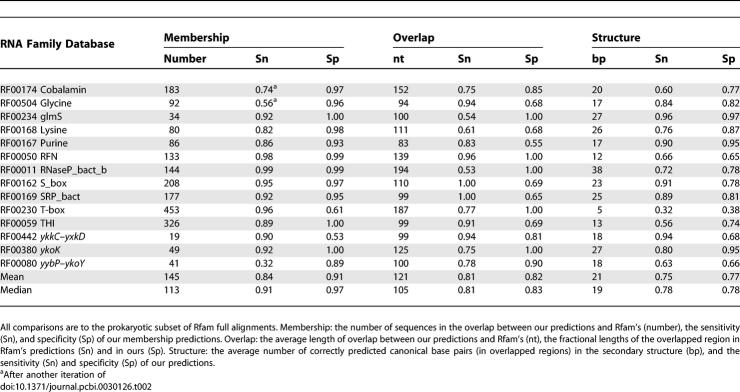
Motif Prediction Accuracy Compared with Rfam

For membership prediction, we achieved an average of 84% sensitivity and 91% specificity. The overlapped regions between predicted motif members and corresponding Rfam members account for 81% of the length of the predicted members, and 82% of the length of Rfam members. In the overlapped regions, the secondary structure prediction has 75% sensitivity and 77% specificity. These results suggest our predicted motif models are very accurate compared with Rfam models, which are learned from the hand-curated seed alignments.

For many riboswitch families, the main differences between our motif models and Rfam models are located in boundary regions. Our predicted motifs tend to include the transcription terminator (if present), which is a stable hairpin followed by a stretch of U's (e.g., Lysine, S_box, T-box). Although transcription terminators are functionally important, the Rfam riboswitch models do not include them. On the other hand, CMfinder tends to miss the closing helix of large multiloop structures (e.g., Cobalamin, *ykoK*). Most other differences are local perturbations such as small shifts or extra base pairs.

As shown in Table 2, we achieved more than 80% membership sensitivity for all families except *yybP–ykoY,* Glycine, and Cobalamin. The predicted *yybP–ykoY* motif differs from Rfam's motif mainly at the multiloop closing helix. Cobalamin and Glycine are two riboswitches with poor sequence conservation (46% and 51% average sequence identity, respectively). While our motifs from the initial full-genome scan may be too specific, sensitivity increases significantly with only a small loss in specificity after another iteration of RaveNnA scan and refinement (unpublished data).

For *ykkC–yxkD* and T-box, we predicted more members than Rfam. The predicted *ykkC–ykxD* motif includes the transcription terminator, which caused false positives in our full-genome scans. These false positives, however, all have much less significant E-values than the true positives, and hence are relatively easy to eliminate by inspection. In contrast, for T-box we believe most “false positives” (with respect to Rfam 7.0) are actually real. Out of 291 members not included in the Rfam full alignment, 127 are upstream of and on the same strand as aminoacyl-tRNA synthetase genes, where most T-box leaders are found, and the others are largely in poorly annotated regions.

### Motifs Not in Rfam

We examined the best-scoring motif (see RNA motif discovery in Materials and Methods and the online supplement at http://bio.cs.washington.edu/supplements/yzizhen/pipeline for details of the motif-scoring function) in each of the top 200 motif clusters. Of these 200 motifs, 116 were deemed unlikely to represent novel ncRNAs: they have covariance model scores less than 40 bits, single hairpin structures, and most were shorter than 30 nucleotides. (Many of these 116 are nevertheless biologically relevant. Many correspond to transcription terminators of upstream genes, and others contain known inverted repeat motifs targeted by DNA binding proteins.) Of 84 remaining motifs, 20 correspond to Rfam families, and 11 to hypothetical transposons. The remaining 53 are candidates for novel ncRNAs. Literature review suggests that many of these candidates are functional. We manually removed the redundant candidates with the same functional roles (for details, see Manual inspection and ribosomal protein leader analysis in Materials and Methods), and present the rest in Table 3.

**Table 3:**
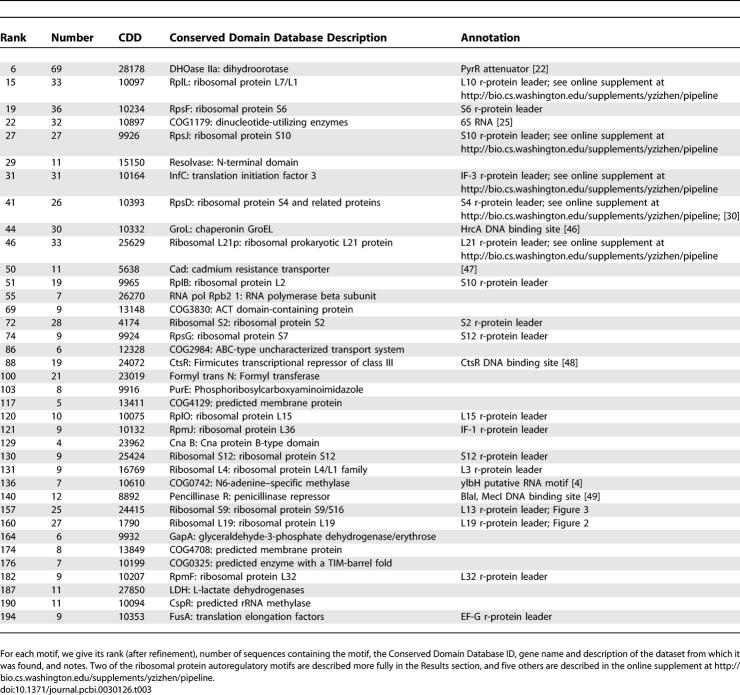
High-Ranking Motifs Not Found in Rfam

**Figure 3:**
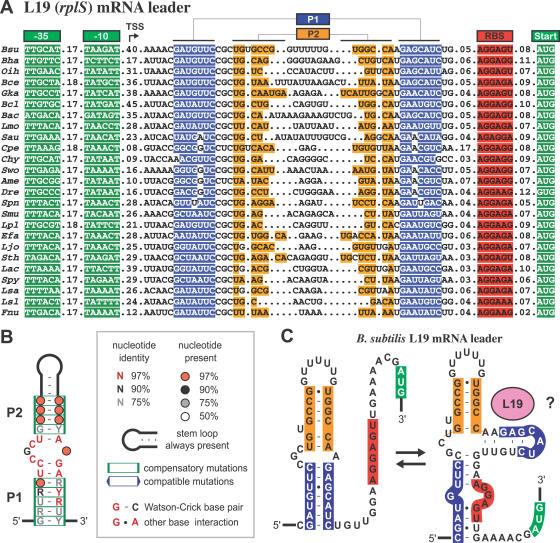
Putative Autoregulatory Structure in L19 mRNA Leaders (A) Sequence alignment of a conserved RNA structure found in the 5′ UTR of Firmicute *rplS* genes. Possible promoter −35 and −10 boxes in genomic DNA are shown, followed by the putative mRNA leader with the predicted secondary structures (P1 and P2), ribosome binding sites, and start codons highlighted. Numbers represent inserted nucleotides that are not shown. The examples shown are representative of 34 total sequences in the complete alignment, available in the online supplement at http://bio.cs.washington.edu/supplements/yzizhen/pipeline. Species abbreviations: *Ame, Alkaliphilus metalliredigenes; Bac, Bacillus sp. NRRL; Bce, Bacillus cereus; Bcl, Bacillus clausii; Bha, Bacillus halodurans; Bsu, Bacillus subtilis; Chy, Carboxydothermus hydrogenoformans; Cpe, Clostridium perfringens; Dre, Desulfotomaculum reducens; Efa, Enterococcus faecalis; Fnu, Fusobacterium nucleatum; Gka, Geobacillus kaustophilus; Lac, Lactobacillus acidophilus; Ljo, Lactobacillus johnsonii; Lmo, Listeria monocytogenes; Lpl, Lactobacillus plantarum; Lsa, Lactobacillus sakei; Lsl, Lactobacillus salivarius; Oih, Oceanobacillus iheyensis; Sau, Staphylococcus aureus; Smu, Streptococcus mutans; Spn, Streptococcus pneumoniae; Spy, Streptococcus pyogenes; Sth, Streptococcus thermophilus; Swo, Syntrophomonas wolfei.* (B) Consensus sequence and secondary structure. Pairs supported by compensatory (when both bases in a pair mutate between sequences in the alignment) and compatible (when only one base mutates but pairing is preserved, e.g., G-C to G-U) are boxed. (C) Structural model of the B. subtilis L19 mRNA leader, showing a possible alternate structure that could be stabilized by L19 binding to repress translation.

#### Annotated motifs.

Several candidates turn out to be known regulatory elements that have been described previously in the literature, including the following.

*PyrR attenuator.* Upstream of CDD 28178, we predicted a PyrR RNA binding site [22], which regulates *pyr* operon transcription by switching between alternative antiterminator versus anti-antiterminator plus terminator structures. The motif we predicted corresponds to the anti-antitermator plus terminator structure, which favors transcription termination. It includes 69 instances in 31 Firmicute species, with two copies per species on average: one copy upstream of the *pyrP* or *pyrR* gene, and one copy upstream of *pyrB.* (Note that the recently released Rfam 8.0 has added a PyrR motif, RF00515, which appears to be in good agreement with our prediction.)

*6S.* This ncRNA binds to *σ^70^* RNA polymerase holoenzyme to globally regulate gene expression in response to the shift from exponential growth to stationary phase. Although 6S has been known in Escherichia coli and close relatives for more than 35 years [23], the corresponding Rfam model (RF00013 6S/SsrS RNA) is confined to *γ*-proteobacteria, and its Firmicute homologs were only identified recently by experimental [24] and computational [25] means; see also [26]. We have discovered 6S in Firmicutes independently in this study. The motif we predict is a partial 6S that includes the most conserved core. (Note that the revised 6S model in Rfam 8.0 now contains Firmicute instances.)

*Inverted repeats.* It is difficult to determine whether a motif with inverted repeats functions at the DNA or RNA level without considering its genomic context. Based on the literature, three single-hairpin inverted repeat motifs in Table 3 appear to be known DNA binding sites for regulatory proteins: HrcA binding sites (rank 44), BlaI/MecI binding sites (rank 140), and hypothetical CadC binding sites (rank 50). (All three are longer and had significantly higher covariance model scores than the 116 removed inverted repeats mentioned above.)

#### Novel ncRNA candidates: Ribosomal protein leaders.

To demonstrate how CMfinder predictions can accelerate the discovery and characterization of new RNA motifs, we present a detailed analysis of two conserved mRNA leader structures that most likely are involved in autoregulation of L19 and L13–S9 ribosomal protein expression. Five additional presumed ribosomal autoregulatory motifs are presented in the online supplement at http://bio.cs.washington.edu/supplements/yzizhen/pipeline.

Many ribosomal protein (r-protein) operons regulate their own expression in E. coli [27,28]. Once enough of a specific r-protein encoded by an operon has been produced (i.e., all of its rRNA binding sites are saturated), excess copies of the protein bind to the 5′ untranslated leader region of its mRNA and induce structural changes that compete with ribosome binding or stall initiating ribosome complexes. This general repression mechanism appears to apply to many r-protein operons, but the specific RNA structures recognized by orthologous r-proteins are generally not conserved between E. coli and other bacterial groups.

For example, the S15 mRNA leaders from *E. coli, Geobacillus stearothermophilus,* and Thermus thermophilus assume different, apparently unrelated RNA structures that all seem to mimic the same rRNA binding site [29]. Similarly, the mRNA binding site of S4 differs between E. coli and *Bacillus* species [30].

Within a bacterial division, the same regulatory structure may be used in many species. Thus, an mRNA leader structure recognized by L4 is conserved in many, but not all, *γ*-proteobacteria [31]. Our comparative analysis using CMfinder is well-suited to recognize r-protein mRNA leader motifs conserved at this taxonomic level. Indeed, it detects the only two r-proteins leader structures that have currently been characterized in Firmicutes (S4 and S15). However, the structure predicted for S4 leaders by CMfinder agrees only partially with a previous phylogenetic analysis of this element based on fewer, exclusively *Bacillus,* species [30]. After manually examining the regions aligned by CMfinder, we predict a consensus structure that is close to the Grundy and Henkin model [30] but has a different pseudoknot (see the online supplement at http://bio.cs.washington.edu/supplements/yzizhen/pipeline). The relatively poor performance of CMfinder on the S4 leaders may be partly due to the clustering of a subfamily of *Lactobacillus* sequences with a slightly different consensus structure from the *Bacillus* sequences. CMfinder performed better on the S15 leader (rank 842), accurately predicting the location and extent of the largest helix-2 feature [32]. Here, it misses only the small adjacent helix-3, and an additional stem that overlaps the open reading frame.

CMfinder also predicts a novel regulatory RNA structure upstream of L19, encoded by the *rplS* gene, in *Bacilli, Lactobacilli, Clostridia,* and *Fusobacteria* species (Table 3, rank 160). In *E. coli,* L19 is expressed as the last of four genes from a polycistronic mRNA [33]. A similar gene order is conserved in some Firmicutes (approximately two-thirds of those with the RNA motif), and there is not an intrinsic transcription terminator between the orthologous upstream *trmD* gene and *rplS* in *B. subtilis.* However, the intergenic distance between *trmD* and *rplS* is typically greater than 100 base pairs in Firmicutes (142 nt in *B. subtilis*) compared with only 41 nt between *trmD* and *rplS* in *E. coli.* Putative promoter −35 and −10 hexamers occur within this intergenic region upstream of each predicted RNA structure (Figure 3A), suggesting that L19 is expressed as a separate transcriptional unit from the upstream genes in Firmicutes.

The putative L19 autoregulatory mRNA structure is a small bulged hairpin (Figure 3B). The length of the terminal P2 stem-loop varies, but the outer P1 helix always has exactly eight base pairs. Most primary sequence conservation occurs in the asymmetric internal bulge and P1 stem. The original CMfinder results include some nonconserved sequences and a spurious stem-loop upstream that are not preserved in all examples. Within the conserved region, CMfinder identifies most of the pairing predicted in our manually refined model.

This RNA structure is always found close to the ribosome binding site (RBS) of the L19 open reading frame. If it is involved in typical r-protein autoregulation, then L19 binding might stabilize an alternate paired conformation wherein the 5′ side of P1 sequesters the RBS to repress gene expression (Figure 3C). Alternately, the predicted P1 stem might only be stable in the presence of L19, and when it forms, its proximity to the open reading frame might prevent translation initiation. Ribosomal protein L19 binds to the large rRNA subunit at the 50S–30S interface. We were unable to identify any homology between the predicted mRNA leader structure and its 23S rRNA binding site in the E. coli ribosome [34], or homologous positions in the B. subtilis ribosome [35], that might suggest a simple regulatory model. It is possible that the predicted regulatory hairpin mimics the structure of the rRNA binding site, or participates in a more complex regulatory mechanism.

**Figure 4:**
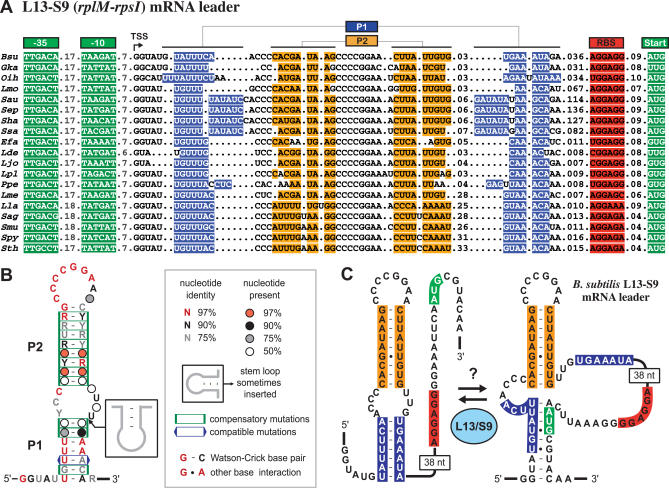
Putative Autoregulatory Structure in L13–S9 mRNA Leaders (A) Sequence alignment of a conserved RNA structure found in the 5′ UTR of Firmicute *rplM–rpsI* operons. The examples shown are representative of 27 total sequences in the complete alignment, available in the online supplement at http://bio.cs.washington.edu/supplements/yzizhen/pipeline. Details are as in the legend for Figure 3 with additional species abbreviations: Lde, Lactobacillus delbruecki; Lla, Lactococcus lactis; Lme, Leuconostoc mesenteroides; Ppe, Pediococcus pentosaceus; Sag, Streptococcus agalactiae; Sep, Staphylococcus epidermidis; Sha, Staphylococcus haemolyticus; Ssa, Staphylococcus saprophyticus. (B) Consensus sequence and secondary structure. (C) Structural model of the B. subtilis L13–S9 mRNA leader, showing a possible alternate structure that could be stabilized by L13 or S9 binding to repress translation.

CMfinder predicts a second novel RNA structure (Figure 4) upstream of the L13–S9 operon, encoded by the *rplM* and *rpsI* genes, in *Bacilli* and *Lactobacilli* species (Table 3, rank 157). There is a strong, near-consensus promoter directly upstream of this motif that defines a conserved transcription start site. The L13–S9 structure is also a bulged hairpin, but it is larger than the L19 motif. There is striking conservation of seven loop nucleotides (CCCCGGA) that are identical in all sequences. Additional conservation occurs in the bulge and within the P1 helix. CMfinder correctly predicts the P2 helix in this manually revised model, and it also identifies the core base pairs in the P1 helix, except in cases where an inserted stem loop occurs in the 3′ side of the bulge.

S9 is a secondary small subunit binding protein, requiring prior S7 binding to associate with 16S rRNA [36]. Most r-proteins involved in autoregulation are primary binding proteins that can bind directly to rRNA, so it seems most likely that L13, a protein that binds to 23S rRNA early in large subunit assembly, recognizes this leader structure. Here again, we were unable to identify any conservation between the rRNA contact sites of L13 and E. coli 23S rRNA or the corresponding sites in B. subtilis 23S that suggest a regulatory model. There is sometimes a significant distance between the putative regulatory RNA structure and the open reading frame. Alternate pairings between the U-rich 5′ side of P1 and a region overlapping the start codon can be devised for many sequences, so it is possible that this alternate conformation is enforced by L13 or S9 binding to the mRNA leader to prevent translation.

In the online supplement at http://bio.cs.washington.edu/supplements/yzizhen/pipeline, we present the full manually refined structural alignments for the above two motifs plus five additional putative r-protein leader regulatory motifs: IF-3, L10, L21, S4, and S10. Based on our experiences with these putative mRNA leader structures, it should be straightforward to define many more candidates for r-protein autoregulatory structures in other bacterial groups with our pipeline. Such studies could illuminate how this form of regulation has been modified and preserved during evolution and would make genomic annotation of noncoding RNAs more comprehensive. Five of these seven putative regulatory RNA elements are now included in Rfam 8.0 (see Accession Numbers).

## Discussion

In this study, we have presented a method for automatically finding *cis*-regulatory RNA motifs in prokaryotes. In a careful test with available sequenced Firmicutes, the method exhibited excellent rejection of negative controls (randomly permuted alignments) and excellent recovery of known, experimentally validated ncRNAs, including most riboswitches known in this bacterial group, as well as RNA elements such as 6S that have only recently been recognized there. Careful inspection and refinement of several novel motifs in ribosomal protein leaders provides compelling evidence that they are indeed conserved structures involved in regulation of these important operons.

In addition, our computational pipeline found dozens of other good RNA motifs that constitute strong candidates for novel functional elements, consistent with the increasing appreciation of the importance of RNA in all living organisms. Finally, our method is sufficiently scalable to be applied to all sequenced prokaryotes. We are in the process of doing so, and preliminary results include several novel riboswitch candidates.

We attribute the power of this pipeline to two key characteristics—a relaxation of the constraints on sequence conservation imposed by most previous methods, and integration of motif inference with genome-scale search. Our method performs motif inference on regions that are not defined by sequence conservation: we search unaligned sequences upstream of homologous genes, instead of multiple-sequence alignments constructed by sequence comparison tools. In addition, both the RNA motif–finding algorithm CMfinder and the RNA homology search algorithms RaveNnA/Infernal exploit structural information. Sequence conservation can be used as well, but is not required. Finally, automatic refinement of motifs to incorporate genome-scale search results has proven to be a powerful component of the pipeline (as in other contexts, such as PSI-BLAST [37]). The integration of these tools enables us to discover RNA motifs with low sequence conservation, and to expand the motif family with remote homologs. For example, the predicted motif for the Glycine Riboswitch has only 35% average pairwise sequence similarity. Remote RNA homologs with appropriate gene context are particularly important, as they are the strongest evidence, short of experiments, that a motif is functional, as well as providing clues to that function.

Future work will seek to strengthen this pipeline by improved exploitation of phylogeny and by an improved scoring system.

Phylogeny is crucial in all comparative genome analysis, without which the concept of conservation is meaningless. It is important in our work because the sequences upon which motif inference is performed are not evolutionarily equidistant, and the significance of conserved nucleotides and compensatory mutations are distance-dependent. Building on the classic phylogenetic likelihood model of Felsenstein [38], Pfold [39] and Evofold [12] use an RNA-oriented phylogenetic model to select from a given multiple-sequence alignment the regions that fit the structural model best. Unfortunately, in our application, neither an alignment nor an evolutionary tree is initially available, and, for our application, use of the corresponding species tree is inadequate in the common case when there are multiple sequences per species. Incorporating phylogeny into motif search is another challenge.

We would also like to improve our scoring scheme. As predicted motifs are subject to expensive manual evaluation and experiments, automatic candidate evaluation to guide resource investment is critical. Our current composite scoring system attempts to discriminate among potential RNA motifs by considering a set of features, including species distributions, structure stabilities, motif sizes, and local sequence conservation patterns. While we can easily recognize motifs that are significant in all these aspects, it is more difficult to order those that are only good by some, but not all, criteria. We have tried to combine the features automatically using machine-learning algorithms such as support vector and logistic regression. However, due to the heterogeneity of the features and limitations of available training data, the results were not as good as our handcrafted composite scoring function. One particular issue is that many of our top-scoring motifs are short single hairpins. They score well because they are widespread, structurally stable, and contain limited but clear sequence conservation. Although short motifs can be functionally important, many do not contain sufficient signal for genome scale homology scans, resulting in false positives that degrade the motif. Other complications include transposons, transcription terminators, DNA–protein binding sites, RNA-polymerase and RNA-ribosome binding sites, etc. The key challenge here is to design a metric that is correctly normalized across various known features and various types of ncRNAs with different sizes, structures, and phylogenetic divergence.

These opportunities for improvement notwithstanding, the approach described in this study has proven itself to be highly effective in discovering noncoding RNA elements in prokaryotes, and promises more discoveries to come.

## Materials and Methods

### Genome sequence and protein homolog data.

We obtained genome sequences from 67 fully sequenced Firmicute species from the NCBI microbial database (RefSeq [40] release 14, 20 November 2005). We first collected amino acid sequences from all annotated protein-coding genes in these species, and categorized them based on NCBI's CDD (version 2.05) [16]. The CDD domain models are curated from various resources, including Pfam, SMART, and COG. In the NCBI microbial database, 92% of all functionally annotated proteins (i.e., with nonhypothetical description field) are assigned to at least one CDD group, as are 32% of “hypothetical” proteins. By definition, all members of a CDD group contain a conserved domain in their protein sequences. A group typically includes both orthologs and paralogs. We assigned proteins to a CDD group using “rpsblast” from the NCBI BLAST package (http://www.ncbi.nlm.nih.gov/BLAST), with an E-value cutoff threshold of 0.01. To reduce redundancy, we removed near-duplicate genomes from analysis. To do this, we created a vector for each complete genome, whose *i*th component was the number of predicted occurrences of the *i*th conserved domain in that genome. We normalized these vectors to have unit (Euclidean) length, and measured their similarity in terms of the projection of one CDD vector onto another (i.e., the dot product between them). Beginning with records assigned the lowest accession numbers, we then assembled a set of genomes by accepting each subsequent genome only when its similarity index with all selected datasets was less than 0.95. After removing redundancy in this way, 44 complete genomes remained for processing in subsequent steps.

We removed CDD groups that contained too few members (four or less), since motif discovery is unreliable on such small groups. We also removed 145 groups with too many members (70 or more), since motif discovery is expensive on such large groups.

### Collecting upstream sequences.

For each gene in a CDD group, we collected a few hundred nucleotides upstream of its start codon, which typically includes both 5′ UTR and promoter sequences. The prevalence of operons in prokaryotic genomes complicates the extraction of the regulatory regions, as the desired regulatory region may be upstream of the entire operon rather than immediately upstream of the selected gene. To handle this complication in a conservative manner, we extracted the noncoding sequences upstream of the gene and upstream of its plausible operon using MicroFootPrinter [41]. Specifically, if the next coding region upstream is in the same orientation and fewer than 100 nucleotides upstream, this short intergenic sequence is included in our sequence dataset, and the same procedure is applied to the upstream gene. This process continues until interrupted either by a coding region in the opposite orientation or an intergenic region longer than 100 nucleotides. Finally, up to 600 nucleotides of the last intergenic region are included in the sequence dataset. After collecting the upstream sequences, we removed redundant sequences (95% sequence identity across 80% of the sequence according to BLAST), and masked regions that match tRNA or rRNA models in the Rfam database.

### Ranking using FootPrinter.

FootPrinter [17] identifies conserved sequence motifs in a set of unaligned homologous sequences using phylogenetic analysis. We scored each FootPrinter motif by the number of motif instances minus the corresponding parsimony score, and scored each dataset as the sum of its top 30 motif scores. The resulting scores are used to rank all datasets. This ranking is performed by MicroFootPrinter [41], a front end to FootPrinter [17].

### RNA motif discovery.

We used CMfinder version 0.2 [14] for RNA motif prediction in unaligned sequences. For each dataset, we produced up to five single stem-loop motifs, five double stem-loop motifs, and used CMfinder heuristics to combine the motifs into more complicated structures if possible.

At various subsequent points, we ranked all CMfinder motifs using a heuristic scoring function that favors motifs with instances in diverged species, stable secondary structure, and local sequence conservation. We used local sequence conservation to discriminate trustworthy alignments with reliable anchors from purely structural motifs (e.g., alignments of single hairpins) that could easily arise by chance, while penalizing global sequence conservation, as highly similar sequences are more likely to be conserved by selection pressure on primary sequence than on structure. We refer to these scores as *composite scores.* The details of the scoring function are described in the online supplement at http://bio.cs.washington.edu/supplements/yzizhen/pipeline.

### Motif postprocessing.

Next, we filtered the motif set to remove poor motifs and combine redundant ones.

Operationally, a “motif” is a covariance model (CM), and a “motif instance” is a sequence that matches the CM with a score above a specified threshold. For each motif, we removed instances with CM score less than ten bits, and removed all but one copy of completely identical instances. Then, we ranked the motifs by composite scores, as outlined above and detailed in the online supplement at http://bio.cs.washington.edu/supplements/yzizhen/pipeline. We further removed motifs with at most four instances and pairwise similarity greater than 0.95, and motifs with composite scores below 50. Afterwards, we selected up to four motifs for each dataset, selected in decreasing score order so that the lower ranking motifs do not overlap significantly with any higher ranking selected motif. By our definition, motif A overlapped significantly with another motif B if the number of nonoverlapping instances of A was less than 30% of the number of overlapping instances, and the average length of the nonoverlapping regions in the overlapped instances of A was less than half of the average length of the overlapped regions. Next, we removed redundant motifs from different datasets. We called motif A redundant with motif B if A overlapped significantly with B and the number of its predicted bases pairs not in B was less than 30% of the number of its base pairs in B. If A and B are redundant with each other, we chose the higher-ranking motif.

Finally, we clustered overlapping motifs as follows. We identified the overlap between motifs according to the genomic coordinates of their instances. One motif was grouped with another if at least half of its instances overlapped, and the overlapped regions are longer than half of the motif length. The motifs were clustered progressively, with high-ranking motifs processed first. We ranked clusters based on their highest-scoring motifs.

### Genome scans for RNA motif homologs.

One of the key strengths of our method is its integration of motif discovery with motif search. Motif discovery is focused on groups of orthologs defined by common CDD membership, since such groups seem likely to be enriched for common *cis*-regulatory elements. However, many *cis*-regulatory elements such as riboswitches will be found near a variety of operons involved in a coherent pathway, which may *not* share a common CDD group. Hence, genome-scale search for additional motif instances is an important component of our approach. Additional instances allow us to construct more accurate motif models, as well as giving insight into potential biological roles for the elements.

Given RNA motifs produced by CMfinder, we searched for additional instances using Infernal CMs [21] accelerated with the ML-heuristic filter [20] implemented in RaveNnA 0.2f. For reasons of speed, two levels of search were used. The initial search database was derived from all 75 finished Firmicute genomes in RefSeq17 (30 April 2006) [40], a total of approximately 200 million nucleotides. Based on sequence annotations, we extracted only intergenic regions for searching, but extended each by 50 nucleotides in each direction to account for common errors in protein-coding gene annotations. The resulting database contained approximately 34 million nucleotides. This small database made it feasible to perform searches for all motifs (averaging 4.8 CPU h per motif), and reduced false positives when compared with the full-genome database. After motif refinement (incorporating hits from this “mini” scan), we performed “full” scans with selected motifs. Full scans examined the prokaryotic subset of the 8 GB RFAMSEQ dataset (version 7.0, March 2005), a total of approximately 900 MB. In particular, comparisons to Rfam (e.g., Table 2) were based on full scans, since Rfam full alignments are also derived from scans of RFAMSEQ. For model refinement, we ran CMfinder on all hits with RaveNnA E-values < 10. E-values were calculated as in [42]. The necessary extreme value distribution calculations dominate the run times for mini-scans, but not for full scans. The refined motif set is again postprocessed and ranked as described above.

### Identifying known Rfam motifs.

To find which of our predicted motifs were already known, we compared them against the Rfam database. Specifically, we BLASTed our motif instances against Rfam full family members (produced by scanning Rfam covariance models on the RFAMSEQ genomic database; see [15]). For BLAST, we used a word size 12, and selected the hits with length greater than 30 nt, E-value < 10, and sequence identify exceeding 90%. These permissive BLAST thresholds resulted in a few isolated hits that we believe to be false positives. These motifs match fragments, each of about 30 bases, of the Rfam RNA-OUT, Intron-gpII, QaRNA, and RNaseP_bact_a families. In general, they are too short, weak, and/or isolated to be compelling, in sharp contrast to the matches reported in Table 1.

### Manual inspection and ribosomal protein leader analysis.

The genomic contexts of the refined motif instances were drawn using the Bio::Graphics modules of BioPerl [43]. For the ribosomal motifs, CMfinder structural alignments were trimmed to relevant regions and manually revised before conducting standard genome scans against the microbial subset of the RefSeq17 database. Hits with the correct genomic context were aligned according to the starting covariance model and manually revised once more to create final sequence alignments (available in the online supplement at http://bio.cs.washington.edu/supplements/yzizhen/pipeline). The Neural Network Promoter Prediction program [44] (version 2.2) was used to predict putative transcription start sites, and programs from the Vienna RNA package [45] were used to examine possible regulatory conformations.

### Online supplement.

Additional datasets and technical details are available at http://bio.cs.washington.edu/supplements/yzizhen/pipeline.

## Supporting Information

Text S1 r-Protein Leader Structural motifs and annotations for predicted ribosomal protein leaders.
(20 KB TAR)

Text S2 Appendix Additional technical details.
(16 KB PDF)

### Accession Numbers

Five of the ribosomal protein leaders discussed in the Results section appear in Rfam release 8.0 (http://www.sanger.ac.uk/Software/Rfam), with the following accession numbers: L10 r-protein leader (RF00557), L13 r-protein leader (RF00555), L19 r-protein leader (RF00556), L20 (IF-3) r-protein leader (RF00558), L21 r-protein leader (RF00559).

## Abbreviations

CDD: conserved domain database
CM: covariance model
ncRNA: noncoding RNA
Rfam: the RNA family database
r-protein: ribosomal protein
