# Supplementary material for: A Computational Pipeline for High- Throughput Discovery of cis-Regulatory Noncoding RNA in Prokaryotes
Source: PLoS Comput Biol. 2007 Jul 6;3(7):e126. doi: 10.1371/journal.pcbi.0030126 (PMC1913097; doi:10.1371/journal.pcbi.0030126)
Supplement: Supplementary file 1 [file pcbi.0030126.sd002.pdf]

A computational pipeline for high throughput discovery of *cis*–regulatory noncoding RNA in prokaryotes: Appendix, additional technical details

Zizhen Yao, Jeffrey Barrick, Zasha Weinberg, Shane Neph,
Ronald Breaker, Martin Tompa and Walter L. Ruzzo

May 10, 2007

1 Scoring CMfinder motifs

We rank CMfinder motifs by a hueristic function over a set of motif features:

- sp: the number of different species in which the motif occurs, measured by distinct Refseq genome IDs.
- mc: average number of motif instances per species. Most riboswitches occur upstream of multiple genes in one species. However, if there are too many motif instances in one species, it is likely to be a repeat element.
- bp: the (weighted) number of base pairs in the consensus structure. To discriminate weak base pair from stronger ones, we weight the base pairs according to the partition function [3, 4], which estimates the probability of forming a base pair averaged over all possible structures.
- lc: local sequence conservation. Most ncRNA families, even ones with low sequence conservation, contain mosaic patterns of local sequence conservation in their sequences. Plausibly, these conserved regions are interaction sites for proteins or other molecules, and are consequently under strong selective pressure. To measure local sequence conservation, we first identify the conserved columns in the given alignment, defined as the columns with more than 70% sequence identity. Then we located all blocks with at least 4 consecutive conserved columns, and computed the total size of all such blocks in a given alignment as lc.
- sid: average pairwise sequence identify. Motifs with high sequence similarity are generally "suspicious", as they are plausibly caused by lack of divergence rather than conservation due to functional importance.

The features bp, lc, sid are computed as the weighted average of all motif instances in a given alignment. The motif instances are weighted based on two criteria. First, CMfinder weights the instances based on their alignment scores, so that poor ones receive low weights. Secondly, we used the Gerstein, Sonnhammer, Chothia algorithm [2] implemented by Infernal [1] to down weight instances with high sequence similarity. The final weight is the product of the two.

The features are integrated in the following ranking function:

$$r = sp \cdot \sqrt{lc \cdot bp/sid} \cdot (1 + \log(mc))$$

We applied the square root and log transformations to prevent a single feature from dominating the overall ranking score.

2 Permuting alignments

To estimate false positive rates for top ranking candidates, we performed a control experiment by running CMfinder on permuted CLUSTALW alignments of CDD sequence datasets. We tried to maintain the

approximate gap distribution of the alignments during permutation by swapping two columns only if their gap patterns have over 80% similarity. Here, we described the gap pattern of each column by a binary vector, which is 0 if the sequence has a gap in the correponding position in the column, and 1 otherwise. To compare the gap patterns of two columns, we merely count the number of matches between the two binary vectors, and the similarity is computed as the ratio of the number of matches over the total number of sequences.

References

[1] Sean R. Eddy. *Infernal User's Guide*, 2003–06. `ftp://selab.janelia.org/pub/software/infernal/Userguide.pdf`.

[2] Mark Gerstein, Erik L. L. Sonnhammer, and Cyrus Chothia. Volume changes in protein evolution. *Journal of Molecular Biology*, 236(4):1067–78, 1994.

[3] I. L. Hofacker, W. Fontana, P. F. Stadler, L. S. Bonhoeffer, M. Tacker, and P. Schuster. Fast folding and comparison of RNA secondary structure. *Chemical Monthly*, 125:167–88, 1994.

[4] J. S. McCaskill. The equilibrium partition function and base pair binding probabilities for RNA secondary structure. *Biopolymers*, 29:1105–19, 1990.
